# Risk factors based on myoma characteristics for predicting postoperative complications following cesarean myomectomy

**DOI:** 10.1371/journal.pone.0280953

**Published:** 2023-03-09

**Authors:** Young-Eun Lee, Suyeon Park, Keun-Young Lee, Ji-Eun Song

**Affiliations:** 1 Department of Obstetrics and Gynecology, University of Hallym College of Medicine, Hallym Sacred Heart Hospital, Anyang, Korea; 2 Department of Obstetrics and Gynecology, University of Hallym College of Medicine, Kangnam Sacred Heart Hospital, Seoul, Korea; Institute for Maternal and Child Health IRCCS Burlo Garofolo, ITALY

## Abstract

**Objectives:**

To evaluate the influence of myoma characteristics on cesarean myomectomy and to demonstrate its additional advantages.

**Methods:**

Retrospective data were collected from 292 women with myomas who had undergone cesarean section at Kangnam Sacred Heart Hospital between 2007 and 2019. We performed subgroup analysis according to the type, weight, number, and size of myomas. Preoperative and postoperative hemoglobin levels, operative time, estimated blood loss, length of hospital stay, incidence of transfusion, uterine artery embolization, ligation, hysterectomy, and postoperative complications were compared among subgroups.

**Results:**

There were 119 patients who had cesarean myomectomy and 173 who had cesarean section only. An increase in postoperative hospitalization and operation time was observed in the cesarean myomectomy group compared to that in the caesarean section only group (mean difference, 0.7 days, *p* = 0.01, 13.5 minutes, *p* <0.001). Estimated blood loss, hemoglobin differences, and transfusion rates were higher in the cesarean myomectomy than in the cesarean section only group. There were no differences in postoperative complications (fever, bladder injury, and ileus) between the two groups. No hysterectomy cases were reported in the cesarean myomectomy group. In subgroup analysis, the larger and heavier the myoma, the higher the risk of bleeding that led to transfusion. Estimated blood loss, differences in hemoglobin, and transfusion rate increased depending on myoma size and weight. A significant increase in postoperative hospitalization was observed in women with larger and heavier myomas. However, there was no statistical difference among the three types of myomas.

**Conclusion:**

In cesarean myomectomy, larger (≥ 10 cm), and heavier myomas (≥ 500 g), were associated with postoperative outcomes, but not the number or type of myoma. The safety of cesarean myomectomy is not inferior to that of caesarean section only, considering its positive effects such as gynecological symptom relief and avoidance of the next surgery.

## Introduction

Uterine leiomyoma is the most common benign tumor and a major cause of morbidity in women in reproductive age [1]. The incidence of myomas during pregnancy has continuously increased in association with a delay in pregnancy. About 10–30% of parturient women with myomas develop complications during gestation, delivery, and puerperium [1, 2]. Some myomas would grow rapidly and during pregnancy because of changes in estrogen and progesterone levels, uterine blood flow, and human chorionic gonadotropin levels. Although most myomas remains asymptomatic, some myomas underwent degeneration and cause severe pain, preterm labor, early pregnancy loss, fetal malformation and placental abruption [1–3].

The first cesarean myomectomy (CM) was performed by Bonney in 1914 [4]. However, CM remains a controversial topic, which is frequently misunderstood as a fatal procedure that eventually results in cesarean hysterectomy. Most obstetricians still hesitate to remove myomas during cesarean section because of concerns about uterine atony, massive postpartum hemorrhage, and subsequent hysterectomy. Recent studies have reported that CM does not cause severe complications and appears to be a safe procedure [1, 2, 5–11]. However, there have been no studies analyzing the outcome of CM based on myoma characteristics, such as myoma number, size, weight, and location. Therefore, this study aimed to evaluate the safety of caesarean section in a CM group compared to group without need of myomectomy (caesarean section only, CSO), and analyzed the effect of various myoma characteristics on the operative outcome in CM.

## Materials and methods

This was a retrospective cohort study of women who had undergone CM or CSO at Kangnam Sacred Heart Hospital, Seoul, Korea, between January 2007 and March 2019. The study protocol was approved by the Institutional Review Board of Hallym University Medical Center (approval no. 2020-02-016). All data were fully anonymized before we accessed them and informed consent was waived due to the retrospective nature of this study.

We included women who had myoma and underwent cesarean section with or without myomectomy at our center. We excluded twin pregnancies, coagulation disorders, uterine anomalies, and placental abruption. The following maternal demographics and characteristics were collected: maternal age, gravidity, parity, gestational age at delivery, body mass index (BMI), and the presence of underlying disease. The characteristics of uterine myomas (number, size, weight, and location) were collected from preoperative ultrasonographic or operation records. The myomas were divided into three categories: subserous and pedunculated (SS), intramural (IM), and submucous (SM). The weight of the myomas was divided into three groups (< 250 g, 250–500 g, and ≥ 500 g). The number of myomas was categorized as single or multiple myomas. The diameter (size) of the myomas was divided into three groups (< 5 cm, 5–10 cm, ≥ 10 cm). In patients with multiple myomas, the diameter of the myoma was defined as the diameter of the largest myoma.

A Pfannenstiel skin incision and a low transverse uterine incision were performed as in a typical cesarean section (CS). Following placental delivery and suture of the uterine incision, myomectomy was performed. A linear incision in the serosa and myometrium was made over the myoma and the myoma was enucleated from its bed. Bipolar cautery was used for hemostasis; however, local injection of vasoconstrictive agents was not used. The remaining defect was sutured to at least two layers with 1–0 absorbable Vicryl (Ethicon, US) sutures. The uterine serosa was sutured using 2–0 absorbable Vicryl sutures. Manual uterine compression and intravenous carbetocin were administered intraoperatively to improve uterine contraction. Oxytocin was administered intravenously postoperatively to enhance uterine involution.

Preoperative and postoperative changes in hemoglobin (Hb) levels, estimated blood loss (EBL) and intra- or postoperative complications were reviewed using electronic medical records. Intra or postoperative complications included fever, bladder injury, ileus, and uterine atony, resulting in uterine artery embolization/ligation or hysterectomy. The operative outcomes of the CM group were compared to those of the CSO group. Changes in outcomes in the CM group were evaluated according to various myoma characteristics (number, size, weight, and type). We further evaluated the subsequent pregnancy outcomes in the CM group.

Continuous variables were summarized as mean ± standard deviation and categorical variables as numbers with percentages. The Mann-Whitney U test, Fisher’s exact test, and Pearson chi-square test were used to compare the two groups. The Jonckheere-Terpstra test was used to compare continuous variables among the three groups (number, size, weight, and type). Statistical analyses were performed using SPSS version 24.0 (SPSS Inc., Chicago, IL) and *p* values <0.05 were considered statistically significant.

## Results

The study population comprised 292 women: 119 underwent CM and 173 underwent CSO. Among total 119 patients in CM group, 98 (82.4%) patients did elective cesarean myomectomy, 16 (13.4%) patients who had not known myoma and discovered during cesarean section by chance, did urgent cesarean myomectomy, and 5 (4.2%) patients did emergent cesarean myomectomy due to failure to labor progression. The baseline characteristics of the study population are shown in Table 1. No statistical differences were observed in the mean maternal age, gestational age at delivery, and BMI between two groups (age, CM, 34.7 vs CSO, 34.4, *p* = 0.61; gestational age, CM, 37.4 vs CSO, 37.4, *p* = 0.49; BMI, CM, 27.8 vs CSO, 28.5, *p* = 0.12). The incidence of nulliparity was higher in the CM group than in the CSO group (CM: 77.3% vs. CSO: 54.9%, p <0.001).

**Table 1:** Clinical characteristics of pregnant women with myomas taken cesarean sections (n = 292).

Clinical characteristics	CM group (n = 119)	CSO group (n = 173)	*p* value
Age (years)	34.7 ± 3.8	34.4 ± 3.7	0.61
Gestational age at Delivery (weeks)	37.4 ± 3.4	37.4 ± 3.2	0.49
Parity			<0.001*
Primiparous	92 (77.3)	95 (54.9)	
Multiparous	27 (22.7)	78 (45.1)	
Body mass index(kg/m^2^)	27.8 ± 4.2	28.5 ± 3.8	0.12
In vitro fertilization (IVF)	8 (6.7)	16 (9.3)	0.44
Previous abdominal surgery	43 (36.1)	104 (60.1)	<0.001*
Cesarean section	15 (12.6)	58 (33.5)	
Myomectomy	9 (7.6)	15 (8.7)	
Adnexa surgery	8 (6.7)	7 (4.1)	
Appendectomy	5 (4.2)	11 (6.4)	
TAC	6 (5.0)	13 (7.5)	
Underlying disease	31 (26.1)	35 (20.2)	0.24
HTN	0 (0)	4 (2.3)	
DM	0 (0)	1 (0.6)	
GDM	12 (10.1)	10 (5.8)	
Preeclampsia	7 (5.9)	7 (4.1)	
Thyroid ds.	12 (10.1)	13 (7.5)	
Indications of cesarean section			
History of cesarean section	15 (12.6)	60 (34.7)	
Malpresentation	21 (17.7)	18 (10.4)	
Large Myoma	18 (15.1)	1 (0.6)	
LGA	5 (4.20)	4 (2.3)	
CPD	5 (4.2)	12 (6.9)	
FTP	7 (5.9)	12 (6.9)	
FDS	2 (1.7)	7 (4.1)	
CDMR	27 (22.7)	10 (5.8)	
Prev. Myomectomy	7 (5.9)	14 (8.1)	
Placental abnormality	2 (1.7)	8 (4.6)	
Underlying disease	5 (4.2)	14 (8.1)	
TAC	5 (4.2)	13 (7.5)	

Data are mean±standard deviation or number (%)

Mann-Whitney U test, Fisher’s exact test, Pearson chi-square were used for comparison of two groups.

*p <0.05, statistically significant.

TAC, Transabdominal cerclage; HTN, Hypertension; DM, Diabetes mellitus; GDM, Gestational diabetes mellitus; LGA, Large for gestational age; CPD, Cephalopelvic disproportion; FTP, Failure to progress; FDS, Fetal distress syndrome; CDMR, Cesarean delivery on maternal request

Table 2 shows the comparison of myoma characteristics between the two groups. The CM group had larger and a greater number of myomas compared with the CSO group (myoma diameter, CM, 6.5 cm vs CSO, 3.7 cm, *p* <0.001; myoma number, CM, 1.7 vs. CSO, 1.2, *p* <0.001). The location and type of the myoma are also described in Table 2. The majority of myoma types in both groups were IM (CM, 61.3%; CSO, 85.0%), followed by the SS types (CM, 33.6%; CSO, 15.0%), while SM had the lowest number of myoma types (CM, 5.0%; CSO, 0%).

**Table 2:** Comparison of characteristics of myomas between CM group and CSO group (n = 292).

Characteristics of myomas	CM group (n = 119)	CSO group (n = 173)	*p* value
Total number of myomas	1.7 ± 1.1	1.2 ± 0.6	<0.001*
Single	70 (58.8)	147 (85.0)	<0.001*
Multiple	49 (41.2)	26 (15.0)	
Number of removed myomas	1.6 ± 1.0		
Weight of removed myomas (gram)	170.9 ± 307.6		
Myoma diameter (cm)			
Largest	6.5 ± 3.7	3.7 ± 1.9	<0.001*
2nd	4.1 ± 2.3	3.0 ± 1.2	0.03*
3rd	4.0 ± 2.3	2.0 ± 0.7	0.01*
Myoma Type			
Subserous & Pedunculated	40 (33.6)	26 (15.0)	<0.001*
Intramural	73 (61.3)	147 (85.0)	
Submucous	6 (5.0)	0 (0)	

Data are mean±standard deviation or number (%)

Mann-Whitney U test, Fisher’s exact test, Pearson chi-square were used for comparison of two groups.

*p <0.05, statistically significant.

The operative outcomes are summarized in Table 3. A statistically significant increase in postoperative hospitalization and operation time was demonstrated in women undergoing CM compared with those who had CSO (mean difference 0.7 days, *p* = 0.01; 13.5 minutes, *p* <0.001, respectively). The estimated blood loss (CM, 724.4 cc vs CSO, 629.5 cc, *p* <0.001), postoperative transfusion rate (CM, 44.5% vs CSO, 17.3%, *p* <0.001) of the CM group were higher than the CSO group. However, there was no difference in differences in Hb level and the incidence of intra-or postoperative complications between the two groups (fever: CM, 29.4% vs CSO, 19.7%, *p* = 0.06; bladder injury: CM, 0.8% vs CSO, 1.2%, *p* = 1.00; ileus: CM, 5.0% vs CSO, 2.9%, *p* = 0.34). No cases of cesarean or postpartum hysterectomy were reported among the 119 parturient women who underwent cesarean myomectomy. S1 Table shows a comparison of fetal outcomes between the two groups. There were no statistically significant differences between the two groups.

**Table 3:** Operative outcomes (n = 292).

Perioperative outcomes	CM group (n = 119)	CSO group (n = 173)	*p* value
Preoperative Hb (mg/dL)	11.3 ±1.8	11.3 ± 1.4	0.95
Postoperative Hb (mg/dL)^a^	9.3 ± 1.5	9.7 ± 1.5	0.06
Hb Differences (mg/dL)	2.0 ± 1.7	1.6 ± 1.3	0.07
Duration of operation (min)	77.0 ± 20.7	63.5 ± 19.2	<0.001*
EBL (cc)	724.4 ± 259.7	629.5 ± 270.0	<0.001*
Length of hospital stay (days)	7.5 ± 2.0	6.8 ± 1.9	0.01*
Transfusion	53 (44.5)	30 (17.3)	<0.001*
Uterine artery embolization	2 (1.7)	2 (1.2)	1.00
Uterine artery ligation	1 (0.8)	2 (1.2)	1.00
Cesarean hysterectomy	0 (0)	4 (2.3)	0.14
Complications	39 (32.8)	35 (20.2)	0.02*
Fever	35 (29.4)	34 (19.7)	0.06
Bladder injury	1 (0.8)	2 (1.2)	1.00
Ileus	6 (5.0)	5 (2.9)	0.34

Data are mean±standard deviation or number (%)

Mann-Whitney U test, Fisher’s exact test, Pearson chi-square were used for comparison of two groups

^a^.Postoperative hemoglobin was measured at 3days after operation

*p <0.05, statistically significant.

Hb, Hemoglobin; EBL, Estimated blood loss

Table 4 summarizes the operative outcomes according to the number of removed myomas (single vs multiple) in the CM group. There was no significant difference in operative outcomes except for the duration of operation (single, 72.6 min vs multiple, 86.3 min, *p* = 0.01). There were no differences in Hb level, EBL, or transfusion rate between the two groups beyond the usual expectations (Hb difference, single, 1.9 mg/dL vs multiple, 2.2 mg/dL, *p* = 0.45; EBL, single, 706.2 cc vs multiple, 763.2 cc, *p* = 0.16; transfusion rate, single, 40.7% vs multiple, 52.6%, *p* = 0.22). In addition, there was no difference in the incidence of intra-or postoperative complications between the two groups (fever, single 29.6% vs multiple 29.0%, *p* = 0.94; bladder injury, single 1.2% vs multiple 0%, *p* = 1.00; ileus, single 49.4% vs multiple 5.3%, *p* = 1.00).

**Table 4:** Comparison of operative outcomes according to number of removed myoma in CM group (n = 119).

Operative outcomes	Single myoma (n = 81)	Multiple myoma (n = 38)	*p* value
Number of removed myoma	1.0 (1 ─ 1)	2.7 (2 ─ 6)	<0.001*
Weight of removed myoma (g)	164.4 ± 352.6	184.9 ± 181.1	0.03*
Preoperative Hb (mg/dL)	11.3 ± 1.8	11.3 ± 1.7	0.76
Postoperative Hb (mg/dL)^a^	9.4 ± 1.5	9.1 ± 1.3	0.33
Hb Differences (mg/dL)	1.9 ± 1.8	2.2 ± 1.4	0.45
Operative time (min)	72.6 ± 18.5	86.3 ± 22.4	0.01*
EBL (cc)	706.2 ± 260.9	763.2 ± 256.2	0.16
Length of hospital stay (days)	7.3 ± 1.8	7.7 ± 2.2	0.60
Transfusion	33 (40.7)	20 (52.6)	0.22
Uterine artery embolization	1 (1.2)	1 (2.6)	0.54
Uterine artery ligation	1 (1.2)	0 (0)	1.00
Cesarean hysterectomy	0 (0)	0 (0)	1.00
Complication	31 (38.3)	14 (36.8)	0.88
Fever	24 (29.6)	11 (29.0)	0.94
Bladder injury	1 (1.2)	0 (0)	1.00
Ileus	4 (49.4)	2 (5.3)	1.00

Data are mean±standard deviation or number (%)

Mann-Whitney U test, Fisher’s exact test, Pearson chi-square were used for comparison of two groups.

^a^.Postoperative hemoglobin was measured at 3days after operation

*p <0.05, statistically significant.

Hb, Hemoglobin; EBL, Estimated blood loss.

The CM group was further analyzed according to the myoma size (Table 5). As shown in Table 5, the larger the myoma, the higher the risk of bleeding that leads to transfusion. The EBL (< 5 cm, 630.4 cc vs 5–10 cm, 733.3 cc vs. ≥ 10 cm 900.0 cc, *p* <0.001), differences of Hb (< 5 cm, 1.7 mg/dL vs 5–10 cm, 2.0 mg/dL vs ≥ 10 cm, 2.5 mg/dL, *p* = 0.04), postoperative transfusion rate (< 5 cm, 26.1% vs 5–10 cm, 45.1% vs ≥ 10 cm, 81.8%, *p* <0.001) were increased depending on the myoma size. Uterine atony was found in three cases; two of these cases that underwent uterine artery embolization occurred in the ≥ 10 cm group and one that underwent uterine artery ligation occurred in the 5–10 cm group. A statistically significant increase in postoperative hospitalization was observed in women with larger myomas (< 5 cm, 6.9 days vs 5–10 cm, 7.6 days vs ≥ 10 cm 8.1 days, *p* = 0.01).

**Table 5:** Operative outcomes according to the myoma size in CM group (n = 119).

Operative outcomes	< 5 cm (n = 46)	5─10 cm (n = 51)	≥ 10 cm (n = 22)	*p* value^a^
Weight of removed myoma (g)	16.4 ± 18.6	136.8 ± 87.9	573.3 ± 536.6	<0.001*
Preoperative Hb (mg/dL)	11.4 ± 1.5	11.3 ± 1.9	11.2 ± 2.0	0.79
Postoperative Hb (mg/dL)^b^	9.7 ± 1.5	9.3 ± 1.5	8.7 ± 1.3	0.01*
Hb Differences (mg/dL)	1.7 ± 1.4	2.0 ± 1.8	2.5 ± 1.8	0.04*
Operative time (min)	73.0 ± 20.9	76.6 ± 19.2	86.3 ± 21.8	0.02*
EBL (cc)	630.4 ± 247.5	733.3 ± 201.7	900.0 ± 314.7	<0.001*
Length of hospital stay (days)	6.9 ± 2.0	7.6 ± 2.1	8.1 ± 1.2	0.01*
Transfusion	12 (26.1)	23 (45.1)	18 (81.8)	<0.001*
Uterine artery embolization	0 (0)	0 (0)	2 (9.1)	0.03*
Uterine artery ligation	1 (2.2)	0 (0)	0 (0)	0.25
Caesarean hysterectomy	0 (0)	0 (0)	0 (0)	1.00
Complication	10 (21.7)	16 (31.4)	13 (59.1)	0.01*
Fever	9 (19.6)	14 (27.5)	12 (54.5)	0.01*
Bladder injury	1 (2.2)	0 (0)	0 (0)	0.25
Ileus	1 (2.2)	2 (3.9)	3 (13.6)	0.08

Data were presented as mean±standard deviation or number (%)

^a^ Linear by linear association or Jonckheere-Terpstra analysis for continuous variables were used to compare 3 groups

^b^.Postoperative hemoglobin was measured at 3days after operation

*p <0.05, statistically significant.

Hb, Hemoglobin; EBL, Estimated blood loss.

Table 6 shows the analysis of the CM group according to myoma weight. The EBL (< 250 g, 687.6 cc vs 250–500 g, 785.7 cc vs ≥ 500 g 1062.5 cc, *p* = 0.01), differences of Hb (< 250 g, 1.8 mg/dL vs 250–500 g, 2.3 mg/dL vs ≥ 500 g 3.5 mg/dL, *p* = 0.02), postoperative transfusion rate (< 250 g, 35.1% vs 250–500 g, 85.7% vs ≥ 500 g 87.5%, *p* <0.001) were significantly associated with the increase of myoma weight. Women with heavier myomas showed significantly longer days of hospitalization (< 250 g, 7.3 days vs 250–500 g, 7.7 days vs ≥ 500 g, 8.6 days, *p* = 0.01).

**Table 6:** Operative outcomes according to the myoma weight in CM group (n = 119).

Operative outcomes	< 250 g (n = 97)	250─500 g (n = 14)	≥ 500 g (n = 8)	*p* value^a^
Number of removed myoma	1.4 ± 0.8	2.4 ±1.7	1.6 ± 1.4	0.08
Preoperative Hb (mg/dL)	11.3 ± 1.8	10.5 ± 1.6	12.5 ± 1.5	0.99
Postoperative Hb (mg/dL)^b^	9.5 ± 1.5	8.3 ± 1.3	9.0 ± 1.3	0.01*
Hb Differences (mg/dL)	1.8 ± 1.7	2.3 ± 1.5	3.5 ± 1.5	0.02*
Operative time(min)	75.2 ± 20.1	82.4 ± 22.8	88.9 ± 21.7	0.06
EBL (cc)	687.6 ± 228.8	785.7 ± 187.5	1062.5 ± 443.8	0.01*
Length of hospital stay (days)	7.3 ± 2.1	7.7 ± 1.0	8.6 ± 1.4	0.01*
Transfusion	34 (35.1)	12 (85.7)	7 (87.5)	<0.001*
Uterine artery embolization	0 (0)	1 (7.1)	1 (12.5)	0.01*
Uterine artery ligation	1 (1.0)	0 (0)	0 (0)	0.64
Caesarean hysterectomy	0 (0)	0 (0)	0 (0)	1.00
Complication	29 (29.9)	10 (71.4)	6 (75.0)	<0.001*
Fever	22 (22.7)	9 (64.3)	4 (50.0)	0.01*
Bladder injury	1 (1.0)	0 (0)	0 (0)	0.64
Ileus	2 (2.1)	1 (7.1)	3 (37.5)	0.01*

Data were presented as mean±standard deviationor or number (%)

^a^ Linear by linear association or Jonckheere-Terpstra analysis for continuous variables were used to compare 3 groups

^b^.Postoperative hemoglobin was measured at 3days after operation

*p <0.05, statistically significant.

Hb, Hemoglobin; EBL, Estimated blood loss.

In addition, the CM group was analyzed according to myoma type (Table 7). There was no statistical difference in operative outcomes between the three types of myomas (SS, IM, and SM). The EBL (SS, 737.5 cc vs IM, 719.2 cc vs SM, 700.0 cc, *p* = 0.85), differences of Hb (SS, 1.7 mg/dL vs IM, 2.2 mg/dL vs SM 1.2 mg/dL, *p* = 0.77), postoperative transfusion rate (SS, 40.0% vs IM, 49.3% vs SM, 1.7%, *p* = 0.82) were not affected by myoma types. In addition, there was no difference in the incidence of each complication among the three types of myomas (fever: SS, 35.0% vs IM, 27.4% vs. SM, 16.7%, *p* = 0.29; bladder injury: SS, 0% vs IM, 1.4% vs SM, 0%, *p* = 0.56; ileus: SS 0% vs IM, 8.2% vs SM, 0%, *p* = 0.15).

**Table 7:** Operative outcomes according to the myoma type in CM group (n = 119).

Operative outcomes	Subserous & Pedunculated (n = 40)	Intramural (n = 73)	Submucous (n = 6)	*p* value^a^
Number of removed myoma	1.4 ± 0.7	1.7 ± 1.2	1.0 ± 0.0	0.72
Weight of removed myoma (g)	128.0 ± 163.8	202.1 ± 370.7	78.0 ± 74.8	0.48
Preoperative Hb (mg/dL)	11.2 ± 2.0	11.3 ± 1.7	11.8 ± 0.2	0.78
Postoperative Hb (mg/dL)^b^	9.6 ± 1.6	9.1 ± 1.3	10.6 ± 1.6	0.52
Hb Differences (mg/dL)	1.7 ± 1.9	2.2 ± 1.6	1.2 ± 1.5	0.77
Operative time (min)	78.1 ± 19.8	77.8 ± 21.5	60.5 ± 6.1	0.14
EBL (cc)	737.5 ± 289.7	719.2 ± 244.8	700.0 ± 268.3	0.85
Length of hospital stay (days)	7.3 ± 1.8	7.6 ± 2.1	6.5 ± 1.4	0.87
Transfusion	16 (40)	36 (49.3)	1 (1.7)	0.82
Uterine artery embolization	0 (0)	2 (2.7)	0 (0)	0.41
Uterine artery ligation	0 (0)	1 (1.4)	0 (0)	0.56
Caesarean hysterectomy	0 (0)	0 (0)	0 (0)	1.00
Complication	16 (40.0)	27 (37.0)	2 (33.3)	0.56
Fever	14 (35.0)	20 (27.4)	1 (16.7)	0.29
Bladder injury	0 (0)	1 (1.4)	0 (0)	0.56
Ileus	0 (0)	6 (8.2)	0 (0)	0.15

Data were presented as mean±standard deviation or number (%)

^a^ Linear by linear association or Jonckheere-Terpstra analysis for continuous variables were used to compare 3 group

^b^.Postoperative hemoglobin was measured at 3days after operation

*p <0.05, statistically significant.

Hb, Hemoglobin; EBL, Estimated blood loss.

To evaluate the risk factors for transfusion in the CM group, we performed multivariate logistic regression analysis (Table 8). Risk factors associated with postoperative transfusion were myoma size (≥ 10 cm) and weight (≥ 500 g). Confounding factors, such as age, preoperative Hb level, prior abdominal surgery history, and underlying diseases, were adjusted. The highest increased risk factors for transfusion were myoma size ≥ 10 cm and weight ≥ 500 g, with adjusted ORs of 13.18 (95% CI 3.45─50.39) and 17.12 (95% CI 1.84─159.60), respectively.

**Table 8:** Multivariate logistic regression model showing the unadjusted and adjusted odds ratios of association between various factors and transfusion in CM group (n = 119).

Variables	Univariable		Multivariable^a^	
	OR (95% CI)	P value	OR (95% CI)	*p* value
Number of removed myoma				
Single	1.0	⋯	1.0	⋯
Multiple	1.36 (0.65–2.830)	0.45	1.39 (0.64–2.99)	0.41
Size of myoma				
< 5 cm	1.0	⋯	1.0	⋯
5─10 cm	2.33 (0.99–5.49)	0.05	2.32 (0.94–5.73)	0.07
≥ 10 cm	12.75 (3.59–45.29)	<0.001*	13.18 (3.45–50.39)	<0.001*
Weight of removed myoma				
< 250 g	1.0	⋯	1.0	⋯
250─500 g	11.12 (2.35–52.59)	0.01*	8.81 (1.75–44.37)	0.01*
≥ 500 g	12.97 (1.53–109.85)	0.02*	17.12 (1.84–159.60)	0.01*
Type of removed myoma				
Submucous	1.0	⋯	1.0	⋯
Subserous& Pedunculated	1.20 (0.36–31.26)	0.29	0.95 (0.26–25.57)	0.42
Intramural	1.58 (0.54–43.71)	0.16	1.38 (0.43–37.19)	0.23

^a^ Age, Pre-op Hb, Prev. abdominal surgery and underlying disease adjusted

*p <0.05, statistically significant.

OR, Odd ratio; CI, Confidence interval.

We also investigated the outcomes of the subsequent pregnancies in the CM group. Among the 119 patients with CM, 54 (45.3%) were lost to follow-up and 42 (35.2%) had no pregnancy at the time of investigation (median time, 5 years; range, 1–13 years). In addition, two women (1.6%) had a spontaneous abortion, and two (1.6%) cases of stillbirth occurred due to preterm labor at 21 weeks of gestation. Nineteen patients (15.9%) underwent additional cesarean delivery after CM. The median interval before conception was 28 months (range: 11–76 days). The median gestational age at delivery was 38+2 weeks (range, 28 weeks─39+1 weeks). The minor complications of the subsequent pregnancy were preterm labor (3 cases), transverse lie (1 case), recurrent myoma (6 cases), and abdominal wall adhesion (6 cases). However, there were no critical complications such as severe intrapelvic adhesion, placental problems (previa, accreta, increta, and percreta), myomectomy scar dehiscence, or rupture during subsequent pregnancy following cesarean myomectomy.

Furthermore, we investigated the long-term follow-up in CSO group. Among total 173 patients, 113 (65.3%) patients were lost to follow-up, 47 (27.2%) patients had symptoms (heavy menstrual bleeding, abnormal uterine bleeding, dysmenorrhea, or low abdominal pain) related to myomas. Among them, 28 (59.6%) patients were prescribed medication or insert intrauterine device (Mirena), 5 (10.6%) patients underwent myomectomy, and 14 (29.8%) patients did hysterectomy.

## Discussion

In this study, CM was safer and more favorable than CSO. Refractory uterine bleeding leading to hysterectomy was not increased by CM, even though myomas in the CM group were larger and more numerous than those in the CSO group. More importantly, our study thoroughly analyzed various characteristics of myomas (number, size, weight, and location) to evaluate risk factors for transfusion in the CM group. There have been no detailed reports of myoma characteristics in CM. We demonstrated that myoma ≥ 10 cm or ≥ 500 g myoma was a significant high-risk factor for transfusion in CM. Interestingly, the myoma number or location did not correlate with transfusion risk following CM.

The safety of CM has been debated over the past few decades. Most obstetricians still hesitate to remove myomas during pregnancy because of concerns about uterine atony, massive postpartum hemorrhage, and uterine rupture during subsequent hysterectomy [12–14]. However, recent studies have suggested that CM can be safely and successfully performed [1, 6, 7, 9, 11, 15, 16]. Our study results are consistent with these recent reports. CM is a safe procedure when performed by experienced surgeons. In this study, none of the patients in the CM group needed further hysterectomy resulting from refractory postpartum hemorrhage or uterine atony, whereas four cases (2.31%) of hysterectomy occurred in the CSO group, with single, small (< 5 cm), and IM types. Furthermore, more cases of uterine artery embolization or ligation were reported in the CSO group than in the CM group. These findings suggest that CM does not commonly cause poor uterine involution or intractable uterine bleeding.

However, the CM group showed longer operation time and hospitalization days and higher transfusion rates. However, mean differences of operation time and hospitalization days between the two groups were 13.5 min and 0.7 days, respectively. Although there were differences between the two groups, the difference was clinically insignificant. Moreover, no massive transfusion, defined as the replacement of 10 units of red blood cells in 24 hours by transfusion, in the CM group was required. In addition, the average number of red blood cell units by transfusion was 2.7 in the CM group and 3.1 in the CSO group (*p* = 0.39). Postoperative day 3 Hb was not different between the two groups, implying that post-transfusion Hb in the CM group was comparable to Hb in the CSO group. Previous reports on CM also demonstrated longer operation time and hospital stay and higher EBL, as in our study [16]. Additional myoma removal and suturing of the myomectomy site naturally increased EBL, operation time, and hospital stay. It is prudent that these marginal differences do not overlook the overall benefits of CM. That is, CM has the benefit of two operations in one, avoiding both the risks and costs of subsequent interval myomectomy. In addition, CSO in women with large myomas has repercussions for the future, including gynecological abnormal heavy bleeding, anemia, and mass effects. Through CM these obvious discomforts can be mitigated and eventually patients’ quality of life can be improved.

In our study, the median length of hospitalization in CM and CSO group were 7.5 days and 6.8 days, respectively. It is different compared with other previous studies [17, 18]. Recent meta-analysis reported that median length of hospital stay of caesarean myomectomy was 3–4 days [17]. In Korea, the length of hospital stay of cesarean section is fixed for 5 or 6 days according to DRG (Diagnosis related group) system and it is very difficult to delay or advance the discharge date by one or two days. For this reason, the length of hospital stay was longer than that of other literatures.

Most importantly, our study is the first to analyze the various characteristics of myomas in the CM group. We analyzed the type, number, size, location, and weight of myomas to evaluate risk factors for postoperative transfusion following CM. Our study demonstrates that the risk of postoperative transfusion was significantly increased in larger or heavier myomas. In particular, a large myoma ≥ 10 cm or greater myoma ≥ 500 g was associated with the highest risk for transfusion. The transfusion risk increased more than 12 times in women with myoma size ≥10 cm or myoma weight ≥ 500 g compared to that in women with smaller or lighter myomas. Large myomas have been referred to as >5 cm in general, [9, 18] but there was no significant difference in postoperative outcomes between the < 5 cm and 5–10 cm groups in our study. However, the risk of transfusion increased in the ≥ 10 cm group. It might be helpful to counsel patients with myoma ≥ 10 cm preoperatively at a higher risk for postoperative transfusion. Moreover, surgeons could warn patients about transfusion risk after surgery when the weight of the myoma specimen is ≥ 500 g.

Some studies have reported that CM should not be performed for certain types of myomas because of surgical complications [14, 19, 20] Roman and Tabsh suggested that CM should be avoided in intramural myomas located in the fundus [19]. Hassiakos *et al*. concluded that myomas located proximal to the fallopian tubes, intramural myomas in the fundus, or myomas located in the cornu of the uterus should be avoided in CM [21]. However, in the current study, there were no statistically significant differences in operative outcomes among the three types of myoma (SS, IM, and SM). In addition, each type of myoma had no impact on the transfusion risk. Thus, it would not be reasonable to determine whether CM outcome depends solely on the myoma type. Experienced surgeons can perform CM safely regardless of the myoma type.

Some studies have reported subsequent pregnancies after CM [20, 22]. Akkurt et al. recently summarized the long-term outcomes of CM in 91 women [20]. Of 91 women, 32 had subsequent pregnancies. All delivered via cesarean section, and none had uterine rupture, while one woman had uterine dehiscence and preterm delivery. The mean interval between CM and subsequent pregnancy was 53.8 months (range: 19–84 months). Three patients (3/32, 25%) demonstrated mild-to-moderate pelvic adhesions in subsequent cesarean deliveries. Adhesion between the omentum and uterus, mild adnexal adhesion, and dense incisional area adhesion were also found [20]. In addition, there were reports of placenta previa in subsequent pregnancies following CM, in which 23 of 119 women had a subsequent pregnancy [20, 22]. Among them, 18 women (18/23, 78.3%) were reported to have subsequent successful term pregnancies, only one woman delivered at 28 weeks of gestation due to preterm labor, only two women had abortions and two had stillbirths at a gestational age of 21 weeks. All 18 term pregnant women underwent repeat cesarean deliveries without any serious complications. No severe intrapelvic adhesions, placenta previa, placenta accreta, increta, myomectomy scar dehiscence, or rupture were observed. Although the number of following pregnancies was small, it might imply that CM performed by a skilled surgeon does not induce critical complications. Further data collection for subsequent pregnancies is needed to elucidate the long-term effects of CM.

This study had several strengths. First, this is the only study to analyze the effect of the diverse characteristics of myomas on the operative outcome in CM. Most previous studies have compared operative outcomes between CM and CSO [14, 23]. A few studies have evaluated the type of myoma in CM, [9, 19] but no studies have thoroughly analyzed myoma characteristics. Second, this is one of the largest studies on CM conducted at a single center. Finally, we demonstrated the pregnancy outcomes following CM. This study reported the safety of CM, the effect of various characteristics of myomas on CM, and subsequent pregnancy outcomes. Our study may be helpful for understanding the overall benefits of CM. The limitations of this study are its retrospective design and the lack of a scoring system for risk stratification. Further large prospective studies are needed, especially regarding long-term outcomes such as gynecological symptom relief (menstruation symptoms, abdominal discomfort, etc.) and subsequent pregnancy.

## Conclusion

When performed by experienced surgeons, CM is a safe procedure. Although the postoperative transfusion risk is higher in CM than in CSO, it is clinically insignificant because massive transfusion is not generally needed. Furthermore, it is reasonable to consider avoidance of discrete surgery, immediate alleviation of myoma-related gynecologic symptoms after delivery, and cost-effectiveness. Most importantly, the risk of postoperative transfusion following CM is correlated with a large myoma ≥10 cm or heavy myoma ≥ 500 g, but not with the number or type of myoma. This might imply that surgeons could be at ease with myoma sizes <10 cm or myoma weights < 500 g. It might be beneficial to counsel patients based on the size of the preoperative sonographic myoma or weight of the postoperative myoma specimen.

## Supporting information

S1 Table Fetal outcomes of pregnant women with myomas taken caesarean sections. (DOCX)
