# Supplementary material for: Risk factors based on myoma characteristics for predicting postoperative complications following cesarean myomectomy
Source: PLoS One. 2023 Mar 9;18(3):e0280953. doi: 10.1371/journal.pone.0280953 (PMC9997914; doi:10.1371/journal.pone.0280953)
Supplement: S1 Table — (DOCX) [file pone.0280953.s001.docx]

## Supporting information

**S Table 1.** Fetal outcomes of pregnant women with myomas taken caesarean sections

| Fetal outcomes | CM group (n =119) | CSO group (n =173) | *p* value |
| --- | --- | --- | --- |
| Neonatal birthweight (g) | 2991.1 ± 695.0 | 2967.4 ± 743.3 | 0.98 |
| Apgar score(1min) <7 | 15 (12.6) | 30 (17.3) | 0.27 |
| Apgar score(5min) <7 | 8 (6.7) | 8 (4.6) | 0.45 |
| NICU admission | 33 (27.7) | 45 (26.0) | 0.74 |

Data are mean±standard deviation or n (%).

Mann-Whitney U test, Fisher’s exact test, Pearson chi-square were used for comparison of two groups.

NICU ; neonatal intensive care unit.
